# Polyolefin Backbone Substitution in Binders for Low Temperature Powder Injection Moulding Feedstocks

**DOI:** 10.3390/molecules19032748

**Published:** 2014-02-27

**Authors:** Berenika Hausnerova, Ivo Kuritka, Davit Bleyan

**Affiliations:** 1 Department of Production Engineering, Faculty of Technology, Tomas Bata University in Zlin, nam. T.G. Masaryka 5555, Zlin 760 01, Czech Republic; E-Mail: bleyan@ft.utb.cz; 2 Centre of Polymer Systems, University Institute, Tomas Bata University in Zlin, Nad Ovcirnou 3685, Zlin 760 01, Czech Republic; E-Mail: kuritka@ft.utb.cz; 3 Polymer Centre, Faculty of Technology, Tomas Bata University in Zlin, nam. T.G. Masaryka 5555, Zlin 760 01, Czech Republic

**Keywords:** binder, polyolefin, carnauba wax, debinding, thermogravimetric analysis, powder injection moulding

## Abstract

This paper reports the substitution of polyolefin backbone binder components with low melting temperature carnauba wax for powder injection moulding applications. The effect of various binder compositions of Al_2_O_3_ feedstock on thermal degradation parameters is investigated by thermogravimetric analysis. Within the experimental framework 29 original feedstock compositions were prepared and the superiority of carnauba wax over the polyethylene binder backbone was demonstrated in compositions containing polyethylene glycol as the initial opening agent and governing the proper mechanism of the degradation process. Moreover, the replacement of synthetic polymer by the natural wax contributes to an increase of environmental sustainability of modern industrial technologies.

## 1. Introduction

Powder Injection Moulding (PIM) has attracted great attention in the production of net-shape, high precision parts for the medical, aerospace and automotive industry in high production volumes due to its low processing cost and time effectiveness. PIM offers clear advantages over other net-shaping techniques, including pressing and casting, for high volume, complex geometry parts of tight dimensional tolerances [1].

During the process, metallic or ceramic powder is mixed with polymeric substances to create a homogeneous feedstock which is moulded into the desired shape after pelletizing. To extract the binder the moulded compacts are treated by chemical (solvent) and thermal routes in the step called debinding. Then, during the final stage the porous products are sintered to a density near the theoretical one.

Such a multi-step processing necessarily evolves often contradictory or inconsistent requirements on the composition of binder system, which must ensure agglomeration-free and uniform distribution of powder particles within feedstock during mixing, and provide the feedstock with moderate viscosity during injection moulding. Then, during debinding it must have steady thermal degradation properties and high yield stress to retain the shape of the product during binder burnout up to early sintering [2]. 

Furthermore, as the PIM technology expands to reactive powders, an additional requirement on binder appears – the need to mix and mould the feedstock at low temperatures in order to diminish the oxidation reactions at elevated temperatures. Additional benefits are energy and material (with protective inert atmospheres such as argon) savings through mixing and moulding steps of the process.

Lack of any of these requirements may result in cracks, voids, distortions, non-uniform shrinkage and warping in the final products [3,4]. Clearly, in order to provide feedstocks tailored to the process demands, multi-component binder systems must be used. A typical binder system includes a thermoplastic polymer (polyolefin) as a backbone, waxes and processing aids [5,6].

In this paper, low density polyethylene (PE) will be substituted with a carnauba wax (CW) and polyethylene glycols (PEG) of different molecular weights. Thermal and combined solvent-thermal debinding of aluminum oxide feedstocks based on PE and CW will be analysed by themogravimetry. 

In a previous paper [7] it has been shown that due to the relatively high hygroscopicity of fine alumina powder it imposes rather sophisticated requirements on binder system. A lot of effort has been done [8,9,10] to investigate the thermal degradation properties of thermoplastic feedstocks based on aluminum oxide occurring as evaporation, thermal degradation and oxidative degradation (in the case of oxygen presence in the atmosphere). Trunec and Cihlar [8] have shown for 60 vol.% feedstock containing ethylene-vinyl acetate copolymer, paraffin wax and stearic acid (SA) in nitrogen (flow rate 3.3 cm^3^·s^−1^, heating rate 10 °C·min^−1^) that high binder loss rates may not allow uniform binder redistribution, resulting in inhomogeneous saturation of the body with binder with porous surface layers and excess binder in the body center, leading to the formation of cracks. 

Krauss *et al.* [9] demonstrated that the solvent (water) extraction of PEG from 55 vol.% alumina feedstock containing polyvinyl butyral as a backbone, PEG and SA is diffusion-controlled, since the weight loss of PEG is square root time dependent. They also developed a mathematical model based on a core-shrinkage mechanism with diffusion in the porous region, and confirmed its good agreement with the experimental data. 

Voorhees *et al.* [10] investigated thermal degradation products of PEG in an alumina feedstock and proposed the concept of the major pyrolysis reactions. The thermogravimetric curves showed that alumina has no effect on PEG/alumina feedstock thermal degradation products.

Finally, it should be mentioned that providing conclusions of general relevance is a rather complicated task, since in the majority of cases the debinding mechanism(s) of one component is dependent on the other components of a binder system, and systematic study of the effect of particular binder components and their concentration in the bulk binder is still missing. The complexity of the compositions used requires detailed studies on each component. Here we report on the role of PEG with different molar mass, polyethylene and paraffin and substitution of these two components by CW from natural sources.

## 2. Results and Discussion

### 2.1. Design of Binder Composition

The binder components employed in the study were selected with respect to the requirements described in the Introduction. The first group of binder compositions (Table 1) contained low density polyethylene (PE). Huang and Hsu [11] studied the effect of high and low density polyethylenes and their blend (50:50) on the properties of 316 L stainless steel feedstocks containing also paraffin wax (PW) and stearic acid (SA) and found out that low density PE results in more favorable flow behavior, however high density PE provides better dimensional stability of the molded part, and thus the combination of both PEs was recommended to gain the maximum benefits. 

**Table 1:** Polyolefin-based feedstocks.

Name	PE	PW	PEG1000	PEG4000	PEG6000	SA	*ɸ_m_*
	wt.%	wt.%	wt.%	wt.%	wt.%	wt.%	wt.%
F1	20	20	10	39	10	1	84.7
F2	10	30	10	39	10	1	85.2
F3	20	20	10	39	10	1	85.2
F4	40	-	20	39	-	1	85.2
F5	40	-	10	25	20	5	85.2
F6a	40	10	-	20	29	1	85.2
F6b							85.5
F6c							85.9
F6d							86.5
F7a	40	10	-	10	39	1	85.2
F7b							85.5
F7c							85.8
F8a	50	-	-	10	39	1	85.2
F8b							85.5
F8c							85.8
F9	20	30	-	20	29	1	85.5
F10	30	30	-	-	39	1	85.2
F11	35	30	-	15	19	1	85.2
F12	30	40	-	12	13	5	85.6
F13	31	33	-	15	16	5	85.2
F14	20	30	10	15	22	3	85.5
F15	25	36	10	10	16	3	85.2
F16	20	10	20	20	27	3	85.2

Hsu *et al.* [12] compared PW, carnauba (CW), polyethylene wax (PEW) and acrawax (AW) in 56 vol.% 304 L stainless steel feedstocks containing 22 vol.% of low density polyethylene (PE) and demonstrated that usage of PW-PE based binders resulted in the highest tensile strength of the sintered parts, however polar waxes (CW and AW) improved the process-ability of the feedstock due to enhanced interactions with powder. Furthermore, AW containing strong polar amide groups and short hydrocarbon chain ends provides the highest carbon content in brown (debinded) parts, and also is less compatible with PE than CW. In order to prevent possible separation and aggregation of PE molecules from the binder during mixing as well as due to the better mechanical properties of CW based feedstocks than AW ones, the former (CW) has been employed as a backbone of the second feedstocks group in this study (Table 2).

**Table 2:** Non-polyolefin based feedstocks.

Name	CW wt.%	PW wt.%	PEG1000 wt.%	PEG4000 wt.%	PEG6000 wt.%	SA wt.%	*ɸ*_m_ wt.%
F17	-	45	-	-	50	5	84.7
F18	-	40	-	15	40	5	84.2
F19	25	30	-	20	20	5	84.5
F20	35	20	10	10	20	5	84.2
F21	40	10	10	12	25	3	84.2
F22	40	10	10	14	25	1	85.2

Waxes and polyethylene glycol are common components of both feedstocks investigated. With low melting temperatures, waxes can be removed at the early stage of debinding consequently creating a network of pores which will promote the thermal degradation of higher molecular weight polymers. Waxes also increase the wetting characteristics of the powder, ensuring uniform coating of the particles.

In this study, the effect of molecular weight of polyethylene glycols is also considered. Yang and Hon [13] and Yang *et al.* [14] studied the influence of low molecular weight polyethylene glycol (PEG) on rheological and debinding characteristics of aluminum oxide feedstock containing PEG, PE wax and SA. Higher content of low-molecular PEG improved the flow-ability of the bulk feedstock. As a processing aid, stearic acid (SA) lubricating and wetting powder particles [15] is used. As it has been reported [16], it plays a critical role in creating homogenous mixture as well as insuring higher rate of binder extraction throughout the network of pores created in body during early stage of thermal debinding.

### 2.2. Thermal Analysis of Feedstocks

The TGA results are presented through the peak degradation temperatures and corresponding mass losses depicted in Table 3. The thermal degradation of highly filled systems proceeds evidently in several steps, however, they are relatively poorly resolved due to the influence of transport processes and the data analysis was elaborated with the aid of numerical derivation. The peak degradation temperature on the negative derivative TGA curve corresponds to the inflexion point in the respective thermal degradation step signalizing thus the highest mass loss rate temperature. In several cases of even less resolved peaks, higher derivatives were calculated and the position of the significant points was refined. If needed, the onset temperatures were obtained from common analytical procedure employing the intersection of the horizontal line at the transition between the distinguishable steps and the tangent in the inflection point [6]. During the debinding stage three main processes are involved: evaporation, thermal degradation and oxidative degradation [17,18]. The low molecular weight compounds or fragments of polymers are mainly evaporated by propagation towards the specimen surface, while high molecular weight components are subjected to thermal degradation [19]. 

**Table 3:** Feedstock thermal degradation peaks in percentage of weight loss.

Feedstock	Peak 1		Peak 2		Peak 3		Peak 4	
	[°C]	[wt.%]	[°C]	[wt.%]	[°C]	[wt.%]	[°C]	[wt.%]
F1	185	0.36	223	11.61	-	-	455	4.98
F2	180	0.19	215	11.92	-	-	447	4.8
F3	175	0.51	215	10.09	-	-	465	5.77
F4	171	0.33	223	10.15	348	4.33	469	2.11
F5	175	0.21	238	9.35	349	4.53	473	2.2
F6a	176	0.15	251	8.82	352	4.97	473	2.18
F6b	184	0.15	234	7.77	338	4.9	472	2.21
F6c	192	0.17	243	8.47	348	4.17	470	1.82
F6d	192	0.14	230	7.8	338	4.42	457	1.95
F7a	189	0.11	242	8.11	356	3.92	462	1.94
F7b	197	0.31	240	8.62	341	4.14	471	2.14
F7c	194	0.19	238	8.21	335	4.34	453	2.1
F8a	181	0.15	232	7.67	348	6.38	454	2.67
F8b	185	0.19	237	7.34	349	5.1	446	2.23
F8c	188	0.18	234	6.9	344	5.3	450	2.34
F9	180	0.23	204	7.51	352	4.48	459	1.29
F10	189	0.22	205	7.63	350	4.6	456	1.54
F11	205	0.32	-	-	354	13.9	462	1.95
F12	201	0.29	245	7.62	348	4.62	460	1.85
F13	207	0.35	251	8.29	349	4.88	455	1.96
F14	176	0.36	219	9.52	-	-	448	5.16
F15	203	0.38	248	9.25	348	4.04	455	1.74
F16	171	0.23	200	9.88	-	-	458	5
F17	174	0.18	208	10.03	-	-	442	4.64
F18	172	0.12	209	12.68	-	-	442	4.51
F19	190	0.16	231	9.07	-	-	460	6.42
F20	194	0.17	245	8.89	341	4.56	458	2.16
F21	189	0.16	-	-	346	13.71	460	2.08
F22	195	0.23	247	8.74	345	4.45	459	1.91

For polyolefins, the thermal degradation occurs by random polymer chain scission [20]. The debinding in an air atmosphere involves oxidative degradation, which acts from the surface towards the center of the specimens, while being limited by the diffusion of oxygen into the binder components and their extraction towards the surface [21]. 

For PE-based feedstocks, the earliest mass loss was exhibited at 171 °C for F4 (40 PE/20 PEG1000/39 PEG4000/1 SA wt.%.) and F16 (20 PE/10 PW/20 PEG1000/20 PEG4000/27 PEG 6000/3 SA wt.%.) compositions by 0.33 and 0.23 wt.% respectively, while the earliest weight drop for CW/PW based compositions took place at 172 °C for feedstock F18 by only 0.12 wt.%.

CW is composed of a mixture of linear chain esters, alcohols, acids and hydrocarbons. It exhibits the smallest linear expansion in temperature range 22–52 °C compared to other waxes [22]. 

For polyethylene glycol (PEG), the polymer degradation process leads to a reduction in molecular weight and to diminution of chain length, resulting from the bond scission in the backbone of the macromolecules as in the case of linear polymers [23]. The TGA data (Table 3) shows that higher overall content of PEG significantly lowers the starting temperature of thermal degradation. 

**Figure 1:**
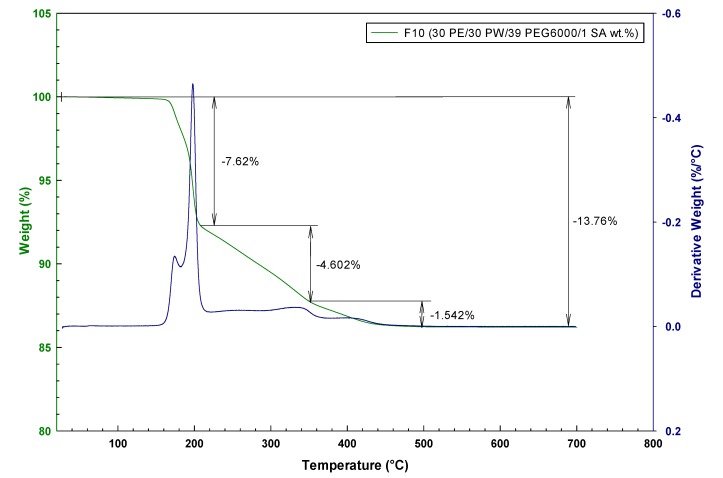
TGA weight loss and corresponding derivative curve for F10 (30 PE/30 PW/39 PEG6000/1 SA wt.%).

**Figure 2:**
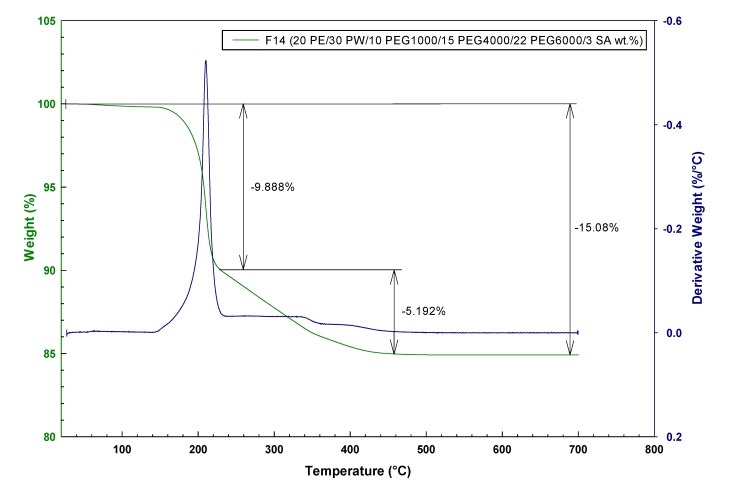
TGA weight loss and corresponding derivative curve for F14 (20 PE/30 PW/10 PEG1000/15 PEG4000/22 PEG6000/3 SA wt.%).

The role of different molecular weight PEG on gradual thermal debinding can be seen from Figure 1 and Figure 2 comparing two contrasting TG curves for PE based feedstock F10 (30 PE/30 PW/39 PEG6000/1 SA wt.%) and F14 (20 PE/30 PW/10 PEG1000/15 PEG4000/22 PEG6000/3 SA wt.%). For feedstock F10 the first mass loss starts at around 189 °C with a sharp drop which is linked to start of the evaporation or degradation of PW and PEG with the lowest molecular mass. Meanwhile, the higher amount of low molecular PEG1000 in feedstock F14 developed a slight slope by the start point at 176 °C. The second stage for both (F10 and F14) feedstocks starting at 205 and 219 °C, respectively, depicts the evaporation of volatile PE degradation products. The weight loss of 13.79 and 15.08 wt.% indicates of a full extraction of the binder system at 456 and 448 °C, respectively.

Typical feedstocks developed by using binder systems with PW and CW as a backbone binders are F17 (45 PW/50 PEG6000/5 SA wt.%) and F22 (10 PW/40 CW/10 PEG1000/14 PEG2000/25 PEG6000/1 SA wt.%), respectively. The feedstock F17 with higher amount of PW and high molecular PEG6000 shows a sharp significant drop of 10.21% at 174 °C. This tendency may lead to cracks and distortions in the sample, and thus must be avoided in further development of binder compositions for the application. The combination of different molecular weight PEG in feedstock F22 developed a slight decrease before the first degradation stage. The first reasonable weight drop starts at 195 °C corresponding to the degradation of PEG blend and passes to a gradual decrease as of the second stage.

In this respect, the second stage represents the extraction of CW, where the exhibited moderate decline is connected with narrow setting range of CW. It shows that the noticeable unwanted rapid drop at the first stage for feedstock F17 can be superceded by proper choice of component’s mass distribution.

The presence of the third peak in –dTGA curves situated in the temperature range 330–360 °C clearly correlates with the presence of reasonable amounts of the high molar mass polymer component in the binder. No peak was observed for compositions having less than 25 wt.% of PE and less than 30 wt.% of CW in the binder. The first step of the thermal decomposition of the binder’s polymer component is manifested in this narrow temperature region regardless of the chemical nature of the polymer used which testifies to the importance of the transport phenomena in the fired body. 

The highest activation energy is needed for decomposition and volatilisation of residual binder mass inside the fired body at the end of the thermal debinding process which is manifested by a decomposition step above 400 °C corresponding to the second decomposition step known for PE and CW. The scatter range of the peak maxima from 440–475 °C is narrow and most likely not influenced by the aluminium oxide filler level regardless of the use of PE or CW, like in the previous step. According to the total mass loss, full binder extraction was achieved for all examined materials.

### 2.3. Thermal Analysis of Water Debinded Feedstocks

The study of TGA results for previously water debinded feedstocks is summarized in Table 4. 

**Table 4:** Water debinded feedstock thermal degradation peaks in percentage of weight loss.

Feedstock	Peak 1		Peak 2		Peak 3	
	[°C]	[wt.%]	[°C]	[wt.%]	[°C]	[wt.%]
F2	210	0.93	225	5.75	487	4.86
F6a	200	2.43	364	11.4	475	1.91
F7a	170	2.48	-	-	477	13.52
F9	170	1.33	250	6.1	480	3.56
F10	220	0.16	229	5.77	490	4.44
F14	199	0.89	220	6.57	494	4.64
F16	175	1.17	252	10.1	449	4.19
F22	208	1.8	230	6.37	467	5.35

The water debinding stage imparts the water-soluble binder components and can be used for two purposes, *i.e.*, as the technological debinding step opening pores serving as channels for volatiles generated during firing. and as the analytical tool for clarification of the role of the aforesaid extractables in the feedstock. A remarkable weight drop was obviously observed for the samples with high vol.% of low molecular PEG binders. The samples with high content of PEG and low content of PW showed the highest water debinding results, and *vice versa* for the samples with high vol.% of PW. PW and PE have the same hydrophobic behaviour but the correlation of debinding efficiency with PW rather than with PE points towards the role of PW as the key blocking agent in the investigated composition framework which may be due a better miscibility of the low molecular compounds with other component of the material system. The same tendency was observed for binders containing the CW although the CW can be considered as less hydrophobic than PE. 

At first sight, the thermal decomposition of the feedstocks was influenced by the water debinding simply by removal of the low molecular PEG component and only three decomposition steps are manifested in the TGA curves. Indeed, the diminishing or disappearance of the low temperature peaks from –dTGA curves as well as depreciation of the observed mass loss steps correlates with the efficiency of the water debinding procedure discussed in the above paragraph. The changes in the mass loss proportions can be explained by the selectivity of the water debinding which removes water-soluble components. Moreover, the peaks seem to be slightly shifted towards lower temperatures indicating thus easier mass removal from the fired body in this temperature region. It can be assumed that the water debinding opened pores into the specimen body by removal of the extractable part of the binder [22].

Thorough analysis of the data in Table 4 in comparison with Table 3 showed that the thermal degradation process appearing in the range between 300 and 400 °C virtually vanishes. As shown in the section above, this decomposition step may be ascribed to the insoluble polymer component of the binder, hence it was not expected that it can be affected by water debinding. One of the effects of the water bath treatment can be not only easier removal of low molecular fractions, but also a faster and better densification of the fired body which can increase the barrier against high molecular binder components decomposition and shift the respective mass loss step to higher temperature.

The high temperature decomposition step between 400 and 500 °C was shifted slightly to higher temperatures (467–485 °C). Such a shift corresponds to an increase of activation energy of the respective process which supports the explanation given in the previous paragraph. The high temperature final step corresponds to the decomposition of residuals entrapped strongly inside the fired body thus requiring a higher activation energy to be removed from the specimen. The mass loss connected with this decomposition step remained unchanged in samples which contained higher amounts of low or moderate molar mass PEG and lower amount of high molecular PEG. The proportion between PE and PW plays important role too. Water debinded specimen F7a which has the highest content of high molecular weight components, *i.e.*, both PE and PEG, showed an enormous increase of mass degraded in this step. A similar correlation was found for the proportion of CW and high molecular PEG. It can be summarized that a balanced proportion of PEG components results into the low high temperature mass loss. According to that the use of PEG with broad molecular mass distribution can be suggested for future experiments. However, it must be pointed out that the aforesaid rule of thumb only works for compounds with balanced proportions between PE and PW or compositions with higher amounts of CW and small additions of PW. The latter conclusion supports the effort to replace synthetic materials by renewable natural stocks. As in the simple thermal debinding, full binder extraction was achieved for all examined water debinded materials.

## 3. Experimental

### 3.1. Materials

**Figure 3:**
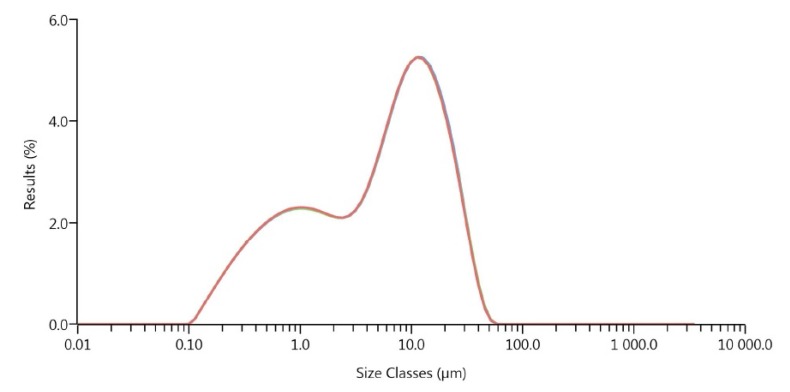
Al_2_O_3_ powder particle size distribution curve (Mastersizer 3000).

High compressible superground aluminum oxide powder (MARTOXID MR70, Albemarle Corp, Barton Rouge, LA, USA) with tap density 2.2–2.4 g/cm³, sintered density 3.74–3.95 g/cm³ and specific surface 6–10 m²/g was used. The the particle size distribution (Figure 3) was measured on a Laser Diffraction Particle Sizing Instrument (Mastersizer 3000, Malvern Instruments Ltd, Malvern, UK).

The properties of polymers serving as a binder components - low density polyethylene (PE), paraffin wax (PW), carnauba wax 2442 (CW), polyethylene glycols differing in their molecular weights (PEG1000, PEG4000, PEG6000) and stearic acid (SA) – are summarized in Table 5. Peak melting temperature was obtained from differential scanning calorimetry (Mettler Toledo DSC1, Greifensee, Switzerland) with heating-cooling rate of 10 °C/min on aluminium pan in nitrogen atmosphere with (60 mL/min) flow. Apparent shear viscosity was determinated with a rotational rheometer Bohlin Gemini CVOR 150 CE/WIN (Malvern Instruments) using a cone-plate geometry (cone angle 2.5°, plate diameter 40 mm) in an air atmosphere. Measurements were carried out at 140 °C and shear rate of 80 rad/s. Other properties were taken from the material data sheets provided by suppliers.

**Table 5:** Characteristic properties of the binder components.

Name	PE	PW	CW	PEG1000	PEG4000	PEG6000	SA
Density [g/cm³] ISO 1133	0.918	0.9	0.97	1.09	1.41	1.21	0.85
Melting Temperature [°C]	108	58	84	32	62	62	70
Molecular Weight [g/mol]	250,000	400	1000	1000	4000	6000	284
Viscosity [kPa.s]	20	2	12	10	72	147	3

### 3.2. Preparation of Feedstocks

A Brabender plasticorder with a mixing chamber of 50 cm³ volume was used for mixing the components. Mixing temperature varied between 100 and 160 °C depending on the composition of mixed feedstock, with the mixing time of 30 min at a speed 20 rpm. In sum 29 feedstock compositions were prepared, with powder loadings (*ɸ*_m_) varying between 84.2 and 86.5 wt.%.

### 3.3. Methods

The thermal degradation properties were studied using a thermogravimetric analyzer TA TGA Q500 (TA Instruments, New Castle, DE, USA) with a platinum pan in an air atmosphere with a constant heating rate of 10 °C/min in temperature range between 30 and 700 °C. Purge gas flow on the balance and sample were 40 mL/min and 60 mL/min, respectively. Feedstock specimens of irregular form were tested. The mass varied between 45 to 58 mg which assured minimization of the specimen weight on the thermogravimetric analysis. In the case of the combined debinding route testing the debinding of water soluble components of binder system components was performed at 60 °C for one hour prior to the thermal debinding.

## 4. Conclusions

From the results it may be concluded that low molecular PEG enhances the early thermal degradation and opens a network of pores in the sample which allows defect free debinding of residual backbone binder. A well balanced ratio between different PEG fractions is required for best performance of the debinding procedure, both for single thermal or two step debinding involving a water bath prior the thermal step. It suggests future use of polydispersive PEG. 

The CW as a backbone ensures gradual and stage followed debinding process, while retaining the shape of the component up to late debinding – early sintering stage due to its very narrow setting range. In next, the application of CW allows one to decrease the amount of PW in the composition.

The thermal debinding process at temperatures above 300 °C is not influenced by the choice of PE or CW but depends exclusively on transport phenomena in the highly filled system. It can be advantageously influenced by the water debinding step application. Besides it may successfully replace the polyethylene as a backbone binder and lower the consumption of PW, so carnauba wax would be the most appropriate choice since it serves as a renewable natural resource which is an outstanding property from an environmental perspective.

## Figures and Tables

**Figure A1:**
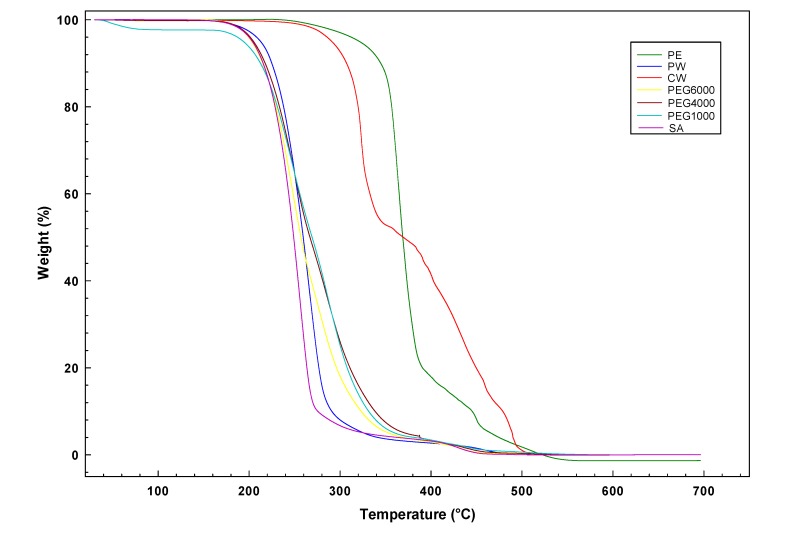
TGA weight loss curves for binders used.
